# Draft Genome Sequence of *Massilia* sp. Strain BSC265, Isolated from Biological Soil Crust of Moab, Utah

**DOI:** 10.1128/genomeA.01199-14

**Published:** 2014-11-13

**Authors:** Alexis C. Bailey, Matthew Kellom, Amisha T. Poret-Peterson, Kathryn Noonan, Hilairy E. Hartnett, Jason Raymond

**Affiliations:** a School of Earth and Space Exploration, Arizona State University, Tempe, Arizona, USA; b Department of Chemistry and Biochemistry, Arizona State University, Tempe, Arizona, USA

## Abstract

*Massilia* sp. BSC265 was isolated from a biological soil crust near Moab, Utah. The strain appears to be capable of chemotaxis and exopolysaccharide synthesis for biofilm adhesion. The BSC265 genome contains a complete dissimilatory nitrate reduction pathway as well as a TCA cycle, making it a facultative anaerobe.

## GENOME ANNOUNCEMENT

*Massilia* sp. strain BSC265 was isolated from a biological soil crust (BSC) collected from a site called Green Butte near Moab, Utah (N38°42′56.2″, W109°41′32″) in May 2009 from a BSC (1). BSCs are microbial communities that form in arid and semiarid environments and supply bioavailable carbon (2), nitrogen (3), and metal-containing compounds (4) to their surroundings via carbon and nitrogen fixation as well as siderophore synthesis (5; K. Noonan, A. T. Poret-Peterson, R. M. Potrafka, A. D. Anbar, F. Garcia-Pichel, and H. E. Hartnett, unpublished data). Here, we present the draft genome of *Massilia* sp. BSC265, a heterotrophic, nitrate-reducing member of *Betaproteobacteria.*

BSC265 was cultured and isolated on BG-11 agar plates modified to detect the production of siderophores (6). DNA was extracted from the isolate using the Ultra-Clean Soil DNA Extraction kit, prepped for sequencing using the Illumina TruSeq DNA HT sample prep kit, and sequenced on 21 June 2014 at the Genomics Core of the Biodesign Institute at Arizona State University using the Illumina MiSeq (Illumina RTA 1/18/54): 850,538 300-bp paired-end reads were generated, resulting in 342.37 Mb of raw sequence data, and 40,525 rho *N*_50_ unitigs with a mean length of 746 bp and remaining reads were assembled into 40 gapless scaffolds and 2 scaffolds with gaps of ≤20 bp using the Celera Whole-Genome Shotgun Assembler version 8.1 for a total genome length of 5,326,161 bp (50× coverage) (7). The scaffolds were screened for DNA contaminants and annotated by the NCBI Prokaryotic Genome Annotation Pipeline, which identified 4,169 genes (8). 16S ribosomal analysis showed 99% identity to the 16S sequence of *Massilia consociata* strain CCUG 58010 from the NCBI 16S Ribosomal RNA Sequences Database using BLAST (9). The G+C content was 65.4%, which is consistent with the *Massilia* genus (10).

Based on the genome of BSC265, it appears to be a heterotrophic member of the BSC microbial community, metabolizing carbon and nitrogen biomolecules produced by other BSC microbes. BSC265 contains the assimilatory nitrogen-compound reduction enzymes nitrate reductase (NasAB) and nitrite reductase (NirBD), the dissimilatory nitrate reduction enzymes nitrate reductase (NarGHIJ), as well as the transcriptional regulators Fnr and Rrf2. The BSC265 genome also contains sulfate metabolism genes; however, the sulfate-reduction pathways are incomplete. BSC265 has genes responsible for chemotaxis, flagellar motility, and exopolysaccharide biofilm synthesis, which facilitate movement toward and adhesion to areas of favorable nutrient conditions. While BSC265 appears capable of anaerobic respiration, it appears to be a facultative anaerobe, containing a complete TCA cycle and terminal cytochrome oxidases.

### Nucleotide sequence accession numbers.

This whole-genome shotgun project has been deposited at DDBJ/EMBL/GenBank under accession number JPXI00000000. The version described in this paper is version JPXI01000000.
